# Studying Membrane Biogenesis with a Luciferase-Based Reporter Gene Assay

**DOI:** 10.3791/920

**Published:** 2008-09-07

**Authors:** Shaochong Zhang, Axel Nohturfft

**Affiliations:** Department of Molecular and Cell Biology, Harvard University; Molecular and Metabolic Signalling Centre, Division of Basic Medical Sciences, St. George's University of London

## Abstract

To study the coordination of different lipid synthesis pathways during membrane biogenesis it is useful to work with an experimental system where membrane biogenesis occurs rapidly and in an inducible manner. We have found that phagocytosis of latex beads is practical for these purposes as cells rapidly synthesize membrane lipids to replenish membrane pools lost as  wrapping material  during particle engulfment. Here, we describe procedures for studying changes in phagocytosis-induced gene expression with a luciferase-based reporter gene approach using the Dual-Glo  Luciferase Assay System from Promega.

**Figure:**
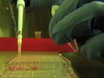


## Protocol

### Cell Culture

Stock cultures of human embryonic kidney 293 (HEK293) cells are grown in 10-cm culture dishes in ‘medium A’, consisting of Dulbecco’s Modified Eagle’s Medium (DMEM) supplemented with a cocktail of antibiotics (100 units per ml of penicillin and 100 µg per ml of streptomycin sulfate) and 10% fetal bovine serum (FBS).
To set up cells for an experiment, the medium is sucked off, and the cells are washed two times with 5 ml of phosphate buffered saline (PBS) solution.
The PBS is then removed and replaced with 0.6 ml of a solution containing 0.25% trypsin.
The dish is incubated at 37˚C for about 2 min until the cells have begun to detach.
5.5 ml of medium A is added to neutralize the trypsin and the cells are counted with a hemocytometer.
Cells are diluted to 320,000 cells per ml and dispensed at 0.1 ml per well into a polylysine-coated 96-well culture plate.
Place the dish into a tissue culture incubator at 37˚C with an atmosphere containing 8.8% CO2 and grow for 24 hrs.

### Transfections

In preparing for transfections, it should be considered how many replicates are to be performed per experimental conditions and what amounts of plasmid will be transfected. We routinely perform all conditions in triplicate and transfect a total of 50 to 100 ng of plasmid DNA per well. Plasmid mixes should include at least a reporter construct expressing firefly luciferase and a control plasmid expressing Renilla luciferase from a constitutive promoter.
Plasmid solutions are pipetted into 1.5-ml microcentrifuge tubes and supplemented with 10 µl per sample of serum-free and antibiotic-free DMEM. For example, if 20 wells are each to be transfected with 50 µg DNA, add 200 µl DMEM to 1 µg DNA.
To the DMEM/DNA solution add 3 µl ‘TransIT 293 reagent’ (Mirus) per µg DNA. Mix by pipetting up & down and let the samples stand at room temperature for at least 10 min.
During this period, remove the media from the 96-well culture plate and replace with 90 µl of fresh medium A.
To each well add 10 µl of the DMEM/DNA/TransIT 293 mixture.
Place the dish into a tissue culture incubator at 37˚C with an atmosphere containing 8.8% CO2 and grow for 24 hrs.

### Phagocytosis

Prepare suspensions containing medium B (a 1:1 mixture of DMEM and Ham’s F12 medium plus antibiotics and 10% FBS) plus 0.75-µm latex beads at zero to 1 mg per ml.
Remove the media from the 96-well culture plate and replace with 0.1 ml of bead suspensions in medium B.
Spin the plate for 2 min at 1000 x g.
The cells are then cultured at 37˚C for 6 to 16 hrs. Depending on the conditions and the promoter used to drive luciferase, increased reporter gene expression can be detected after 1 to 3 hrs.
In same cases it may be necessary to incubate the cells with beads for 30 to 60 min, then do 2 washes with PBS to remove unincorporated beads, and add fresh media before incubating the cells for additional periods of time.

### Dual-GloTM Luciferase Assay

Note: our protocol for using this kit differs in some respects from that recommended by the manufacturer. It has been modified to reduce the amount of reagents required per sample.
Prepare a solution of 1 M DTT, divide into multiple small aliquots and store at -20˚C.
Prepare a lysis buffer containing 20 mM Tris-HCl (pH 7.8), 10% (v/v) glycerol, and 0.5% (v/v) Triton X-100. Prior to use, aliquot an amount needed for the experiment and add 1 µl per ml of 1 M DTT plus protease inhibitor cocktail to a final concentration of 0.5 to 1x. Do not reuse the leftover DTT solution.
When using the Dual-GloTM kit for the first time, prepare the (firefly) Luciferase Reagent by transfering the entire contents of one bottle of Dual-GloTM Luciferase Buffer (provided with the kit) to one bottle of Dual-GloTM Luciferase Substrate (provided with the kit). Divide the unused Luciferase Reagent into 10-ml aliquots and store at -20˚C.
Remove the 96-well plate from the tissue culture incubator and place it on ice. Remove the media by aspiration, add 40 µl per well of lysis buffer, and then leave the plate on ice for 30 min.
In a white, opaque 96-well plate (OptiplateTM-96, Perkin Elmer catalog number 6005290) aliquot 15 µl per well of Luciferase Reagent, and then add 15 µl of cell lysate.
Incubate the plate at room temperature for 10 min and then read the luminescence in a microplate reader.
Immediately before use, prepare an appropriate amount of Renilla luciferase substrate solution by adding 1 part Dual-GloTM Stop & Glo® Reagent (provided with the kit) to 100 parts Dual-GloTM Stop & Glo® Buffer (provided with the kit).
To each well add 15 µl per well of the Renilla luciferase substrate solution, incubate at room temperature for 10 min and then measure the luminescence again.

